# Gas Flow to Enhance the Detection of Alpha-Induced Air Radioluminescence Based on a UVTron Flame Sensor

**DOI:** 10.3390/s18061842

**Published:** 2018-06-05

**Authors:** Anita J. Crompton, Kelum A. A. Gamage, Steven Bell, Andrew P. Wilson, Alex W. Jenkins, Divyesh Trivedi

**Affiliations:** 1 Engineering Department, Lancaster University, Lancaster LA1 4YW, UK; 2 School of Engineering, University of Glasgow, Glasgow G12 8QQ, UK; kelum.gamage@glasgow.ac.uk; 3 Nuclear Metrology Group, National Physical Laboratory, Teddington TW11 0LW, UK; steven.bell@npl.co.uk; 4 Independent Researcher; andy.wilson1962@talktalk.net; 5 Characterisation, Inspection & Decontamination Group, Sellafield Ltd., Cumbria CA20 1PG, UK; alex.jenkins@sellafieldsites.com; 6 The National Nuclear Laboratory, Warrington WA3 6AE, UK; divyesh.trivedi@nnl.co.uk

**Keywords:** UVTron flame detector, alpha detection, alpha-induced air radioluminescence, alpha imaging, nuclear decontamination and decommissioning, gas scintillation

## Abstract

In many field applications where alpha-induced radioluminescence (or so-called UV fluorescence) could potentially be used for stand-off detection of alpha-emitting materials, it may not be possible to create a fully purged gas atmosphere. Hence, an alternative gas delivery method to utilise the radioluminescence enhancing properties of gases has been investigated, with the novel results from this presented herewithin. A solar blind ultraviolet C (UVC) sensor (UVTron R9533, Hamamatsu, Japan) has been used to detect changes in the signal in the UVC wavelength range (180–280 nm), where gases of Ar, Xe, Ne, N_2_, Kr, and P-10 were flowed over a 6.95 MBq ^210^Po source using a narrow diameter pipe close to the source. In comparison with an air atmosphere, there was an increase in signal in all instances, the greatest being the flow of Xe, which in one instance greater than doubled the average counts per second. This increase in signal could prove beneficial in the design of a stand-off alpha detector to detect the very small UVC radioluminescence signals from alpha-emitting materials found in nuclear decommissioning environments.

## 1. Introduction

Due to the short range of alpha particles in air (approximately 5 cm depending on energy), detectors which require direct interaction to detect alpha radiation are required to be operated in very close proximity to any surface under examination. This means that detection of alpha-emitting contamination is time-consuming and places personnel in close proximity to potentially hazardous materials, including the possibility of exposure to a mixed radiation field as is found in many real world nuclear environments.

As alpha particles travel, they transfer energy to and ionise the air. The relaxation from this excited state causes the emission of optical photons. These photons have a much greater mean free path than the original alpha particle and hence provide a possible opportunity for stand-off alpha detection. This radioluminescence is mainly in the 300–400 nm wavelength range [1,2]. However, in this range, there is much interference from background light, either from the sun or from indoor lighting, making the detection of this radioluminescence problematic. Stand-off detectors trialled to date have usually operated in darkness or special lighting conditions [1,3,4,5,6,7,8,9,10].

Other work has looked at the emissions in the UVC (ultra violet C) wavelength range (180–280 nm), which although lesser in intensity has much lower background levels with which to contend. UVC from the sun is mainly stopped in the atmosphere, meaning little reaches the surface of the earth, and UVC is generally not emitted by indoor lighting as it can be hazardous to eyes and has no practical benefit as it is well outside of the visible light spectrum [11]. This has meant that stand-off alpha detection in the UVC wavelength range has been investigated with some positive results for detection in daylight conditions [12,13].

In light of the low signal intensity of UV, and especially UVC photon emissions even from a relatively active alpha source, a means to enhance the signal could reduce the burden on any detector system in terms of how sensitive it would be required to be to detect alpha-induced radioluminescence in field operation. This would provide a better chance of detecting a low activity source or in bright daylight conditions, and to this end, research has been carried out into the effect of a gas atmosphere on the level of radioluminescence, including in the UVC wavelength range. Section 2 looks at previous research to date, with the remainder of sections detailing the results of recent experiments using a flow of gas.

## 2. Gas Atmosphere Influence on Radioluminescence Spectrum and Yield

Nitrogen makes up approximately 78% of the composition of air and is the main cause of radioluminescence in a dry air atmosphere. The other main constituent of air, oxygen at approximately 21%, has been shown to quench radioluminescence [14,15]. The remainder comprises small proportions of other gases (approximately 1% argon, 0.04% carbon dioxide, and fractional quantities of neon, helium, methane, and other gases). To determine if changes in the atmosphere surrounding an alpha source have an effect on the radioluminescence yield, experiments using a purged gas atmosphere have been carried out.

Some researchers have compared the alpha-induced radioluminescence readings of two PMTs in an N_2_ atmosphere, one sensitive in the 160–650 nm wavelength range, the other 300–650 nm, and found little difference between the two [14]. From this they concluded that N_2_ radioluminescence occurs mainly in wavelengths of above 300 nm. This held true for N_2_, dry air, O_2_, carbon dioxide (CO_2_) and methane (CH_4_) atmospheres. O_2_, CO_2_, and CH_4_ produced significantly less radioluminescence then N_2_ and dry air and quench radioluminescence when mixed with N_2_ due to the excited states of these gases not decaying through the emittance of photons. The amount of quenching depends on the composition of the secondary gas and the percentage of that gas, as can be seen by decreases in the radioluminescence yield in an N_2_/O_2_ atmosphere when the percentage of O_2_ is increased. In an N_2_ atmosphere, there is however less oxygen quenching on the NO lines, which are in the UVC wavelength range [15].

Although this increase in radioluminescence due to N_2_ takes place mainly in the ultra violet A and ultra violet B (UVA and UVB) regions (300–400 nm), other gases have the potential to fluoresce in the UVC wavelength range, and these may prove beneficial in detecting alpha particles through UVC photon emissions. There are difficulties in examining luminescence of rare gases due to them fluorescing in the Vacuum Ultraviolet (VUV, 10–200 nm) wavelength region [16]. However, several of these gases also fluoresce in the UVC wavelength region, albeit not as strongly.

**Figure 1:**
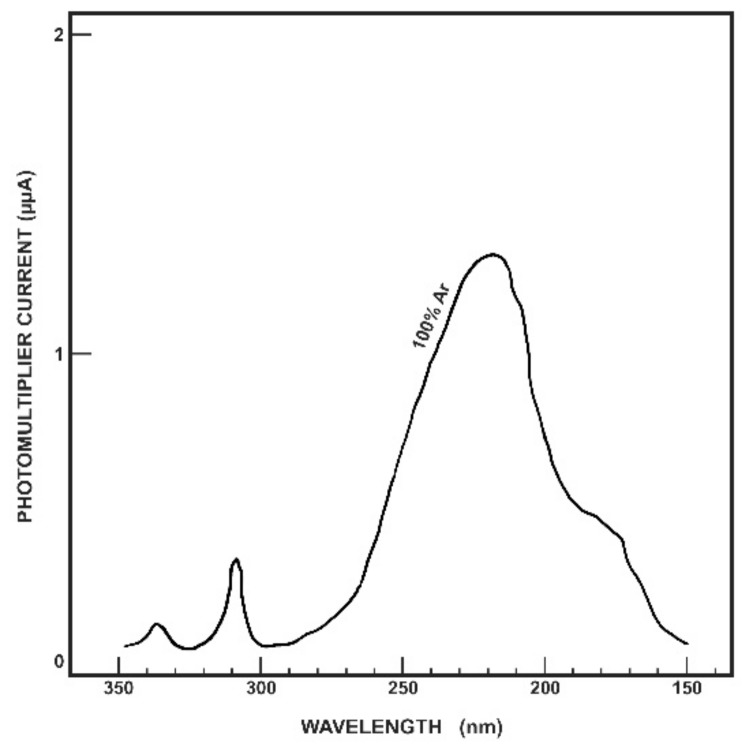
Radioluminescence spectrum of 100% argon. Figure reproduced from [17].

Argon has been shown to luminesces in the VUV and UVC wavelength region, in three distinct wavelength bands, with maxima at 130, 180, and 220 nm [17]. The 220 nm peak can be seen in Figure 1, which also shows the peaks in the UVA and UVB wavelength ranges.

The highest intensity of these maxima was found to be 220 nm, with 180 nm the lowest, although the 180 nm band was less easily quenched, and the intensity of radioluminescence was less dependent on pressure. The 220 nm Ar peak value of 45 (in arbitrary units) is greater than the 236 nm peak produced by N_2_, which reached only 32 (in arbitrary units). Increasing the pressure of an Ar atmosphere (between 0.07 and 2.63 atm) leads to an increase in radioluminescence in the 170–280 nm wavelength range, with this being more rapid between 200 and 280 nm.

However, others found an Ar atmosphere fluoresced with a distinct peak at 127 nm, with no other peaks in the 115–300 nm range, but a small, gradual rise between 200 and 270 nm [16], although this has been seen to peak at approximately 250 nm before starting to decline [15]. They identified the VUV peak as due to the recombination of ions with electrons, which in their later work they determined produced one VUV photon per recombination [18]. They suggest that in the 200–270 nm region radioluminescence is due to the third continuum, and that the mechanism of this has as yet no satisfactory explanation. They also found peaks of 150 nm for Kr and 175 nm for Xe, just outside the UVC range, with a Gaussian distribution of width 13 nm and 15 nm (FWHM) respectively. The Ar 127 nm peak also had a Gaussian distribution with a width of 11 nm (FWHM). They also examined the travel of an alpha particle in different gases and calculated the range of alpha particles is approximately 48 mm in Ar, 34 mm in Kr and 25.2 mm in Xe [16]. They also discovered that increased pressure led to an increase in recombination radioluminescence, and that this was greater in Xe than in Ar or Kr.

In alpha-induced ionisation within gases, the average energy per ion pair was found to be due to the stopping power of the gas and independent of alpha particle energy for hydrogen, helium, N_2_, Ar, and nitrous oxide [19,20]. Others found that the average energy value per ion pair in Ar for alpha particles of different energies was consistent for particles over 5 MeV [21]. This would suggest that the gas medium rather than alpha energy is more important in determining radioluminescence wavelength and therefore is relevant in potentially increasing radioluminescence intensity in the UVC wavelength range.

Kerst et al. investigated the effect of N_2_ on radioluminescence in the UVC wavelength range [22]. They tested a ^210^Po source in an N_2_ purged atmosphere and found increased radioluminescence in the sub 300 nm wavelength range due to an increase in nitric oxide (NO) luminescence. Alpha emitters in air produce electronically excited nitric oxide molecules, and the presence of N_2_ enhances this radioluminescence to detectable levels [22].

Although Ar is readily available at nuclear facilities and certainly fluoresces in the UVC wavelength region, it may be more beneficial to consider an alternative which produces a significant peak in a wavelength more suited to existing UVC detectors—for example the UVTron which has a wavelength detection range of 185–260 nm. Xe peaks at a higher wavelength than Ar and may be more suited to UVC detection enhancement.

## 3. Materials and Methods

Based on the prior research laid out in Section 2, an experiment was devised which would test the effect of different gases on alpha-induced radioluminescence in a method which could potentially be used in the field. As a flow over the source does not depend on a purged gas atmosphere, it may be more suited to a wider application in the field, where the contamination could be in a large space or spaces containing personnel. A flow of gas within a purged atmosphere when researching the use of protons to excite gases has been found to be preferable to a static atmosphere as it increases the intensity of the radioluminescence [23]; hence, the use of a flow is not unprecedented for research in this area.

Five noble gases were selected for the tests; N_2_, Xe, Ne, Kr, and Ar. These were selected as the most likely to fluoresce in the UVC wavelength range. The results can also be compared to previous research which was carried out with several of the gases. P-10 (90% Ar, 10% methane) was also tested as this gas mixture is utilised for its ionising properties.

**Figure 2:**
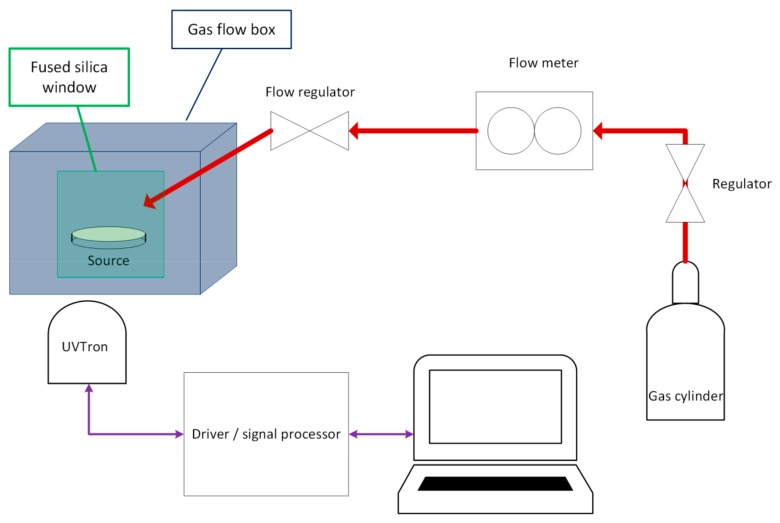
Schematic of equipment set up.

Experiments were carried out at the National Physical Laboratory in Teddington, Middlesex. The set up for the experiments was as follows (see Figure 2).

**Figure 3:**
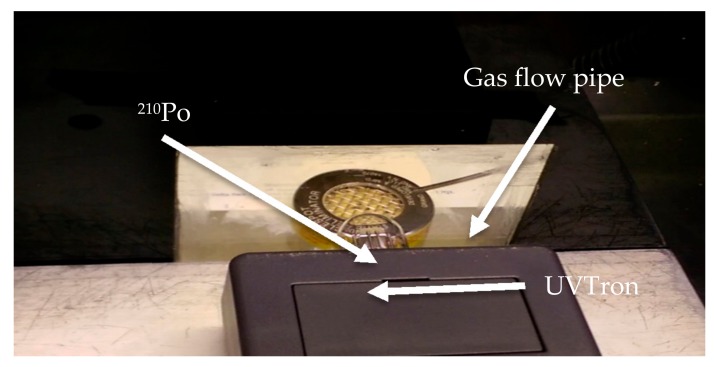
Photograph showing the ^210^Po source inside the gas flow box (silver disk with mesh surface and yellow edge), with the gas flow pipe above and to the left. The UVTron (small glass bulb) is external to the box and is attached to the grey box in the foreground which houses the detector electronics.

A sealed ^210^Po source with activity of 6.95 MBq (at time of experiment, 29 August 2017) was placed inside a black Perspex box of dimensions 260 × 234 × 230 mm (see Figure 3). ^210^Po was selected for the experiments as it decays through alpha emission only and therefore produces negligible other emissions to affect the sensor reading. The box has a 75 × 75 mm window of 2 mm thick synthetic fused silica (Spectrosil©). Fused silica is preferable to ordinary glass as it allows UVC to pass with minimal attenuation (less than 10%) [24]. This window also prevented alpha particles being directly incident on the sensor. A lid was placed on top of the box at the commencement of and throughout each experiment to prevent the fume hood extractor system from removing the gas before it reached the source. The chamber was not gas tight, and some loss/exchange of gas would have occurred.

The UVTron sensor was placed outside of the box approximately 20 mm from the source (see Figure 3). The UVTron (R9533, Hamamatsu Photonics, Hamamatsu, Japan) is sensitive in the 185–260 nm range. It is designed to detect flames or corona discharge but has been verified in its use as a sensor to detect alpha-induced air radioluminescence and has a very low background count in laboratory lighting conditions [13]. The UVTron was used with the off-the-shelf driver circuit (C10807, Hamamatsu Photonics, Hamamatsu, Japan) which both powers the UVTron and conditions the output signal. An Arduino Uno was used to count the pulse outputs from the driver and relay this to the laptop. The driver outputs one pulse per incident photon, up to a maximum of 40 counts per second (cps). This is sufficiently fast for alpha radioluminescence counting for this source activity and separation distance, where less than 10 photons per second are incident on the detector within the wavelength range [13]. The sensor has a peak sensitivity at approximately 210 nm, with an equal increase and decrease in sensitivity on either side of this wavelength between 185 and 260 nm, which are the limits of the wavelength range of the UVTron and therefore at which the sensitivity is 0.

The UVTron was used in these experiments for several reasons. It is solar blind and has a very low background count and so can be used in normal laboratory lighting without the need for filtering or other light-attenuating technology. It is a relatively low-cost, robust, off-the-shelf sensor suited to inclusion in a field operable detector and fitting with the experimental main aim of testing a system which may be more suited for field operations.

Gas was flowed over the source from close proximity in an air atmosphere using a small flexible pipe of 1 mm bore diameter (see Figure 3). At the end of the experiment, the lid was removed from the gas flow box and the fume hood extractor system was switched on to extract the gas. Following this, the pipes were purged with the new gas, and the flow was regulated, with a steady flow reading established before the next experiment was undertaken. The gases and flows are listed in the table below (Table 1).

Group 1 experiments consist of those carried out and already reported on by Crompton et al. [13]. 

## 4. Results

### 4.1. Background

In order to allow for any effect from background lighting, for each of the experimental sessions, a background count was taken. The experiment was set up as detailed in the above section but without the presence of the source. The background had been shown to be very low, with average cps of 2.2 ×10^−3^ ± 0.7 × 10^−3^ for group 1 [13]. For groups 2 and 3, the background was 1.48 × 10^−3^ ± 0.53 × 10^−3^. The experiments were carried out in the same laboratory with standard commercial strip (fluorescent) lighting which remained on for the duration of all experiments. The experiments were conducted a number of weeks apart, and therefore differences such as location of the equipment, temperature, humidity etc. in the laboratory between the two dates is most likely the cause of the difference in the background recorded for groups 2 and 3 compared to those for group 1 [13].

### 4.2. Air Atmosphere Results

The experiment was first carried out with the source in place in an air atmosphere to provide a baseline to compare the results of different gas flows. The gas flow experiments were carried out in three groups, and at the commencement of each, the count in air was taken for comparison. This was done to ensure that any changes in the position or orientation of the equipment could be allowed for in the comparison between the air and gas results. The counts in air were average cps of 0.3282 ± 0.0032 for group 1 [13], 0.4106 ± 0.0107 for group 2, and 0.4503 ± 0.0028 for group 3.

### 4.3. Gas Flow Results

**Figure 4:**
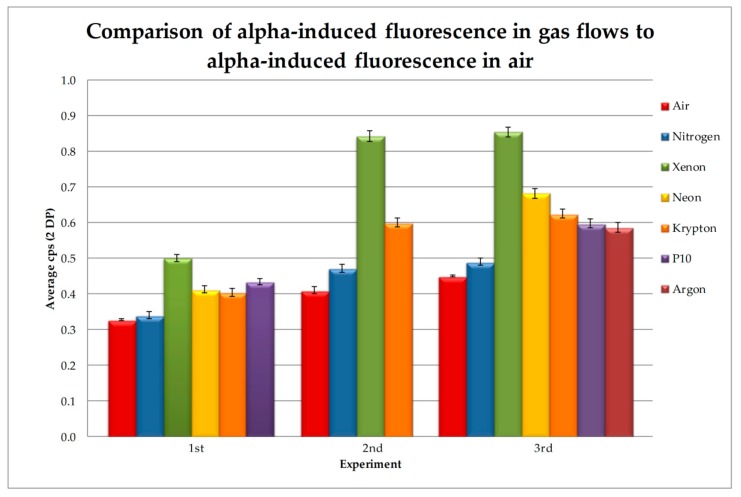
Showing the cps for each of the gas flows in each of the three experiment groups (note: not all gases were tested in the second group due to time constraints) comparing the results of those in the first group reported by Crompton et al. [13] with two following groups.

**Figure 5:**
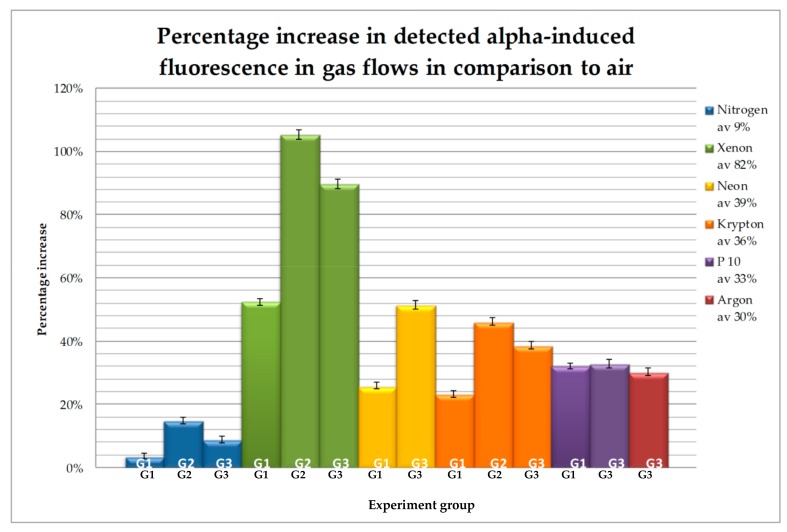
Showing the increase in cps for the different gas flows as a percentage of the air atmosphere results.

The results of the gas flow experiments carried out on this occasion in comparison with those already reported [13] are shown in Figure 4 and Figure 5, and Table 2. Figure 4 shows the average cps for each of the gases for each of the three groups of experiments (note, not all gases were tested in all groups due to practical issues). Figure 5 shows the increases for the different gases as compared to the air atmosphere, which are shown as percentages increase from the average air cps. Table 2 lists the data for further clarity.

As can be seen from Table 2, the greatest increase was seen with a flow of Xe, which increased the radioluminescence between 52% and 105%, with an average over the three experiments of an 82.5% increase. Ne provided the next greatest increase, with an average of 39% over the two experiments, which was close to the average increase of Kr of 36%. P-10 gave the next greatest increase, averaging 33% over the two experiments, with Ar slightly lower than this at an average of 30%. P-10 gave the more stable result over the two experiments, with a difference of less than 0.7% between the two readings, giving it the second highest average in the group 1 readings.

Group 1 has the lowest increase across all gases in comparison to groups 2 and 3. This is most likely due to slight differences in the set-up—for example, the position of the gas flow pipe in relation to the source which may have affected the make-up of the atmosphere within the MFP of the alpha particles emitted from the source, for example by increasing quenching by oxygen. 

## 5. Discussion and Conclusions

From these results it can be seen that all the gases increased the radioluminescence detected to some degree. This is most likely due to the replacement of O_2_, which quenches radioluminescence, in the scintillation zone of the alpha particles.

The increase in the cps produced by a Xe atmosphere, especially as the detection rate may be as much as doubled, does indicate that the use of a flow of this gas could be useful in the development of an alpha detection system. As the alpha signal is low in the UVC wavelength range, but in light of this range having the least background interference, the enhancement of the signal could reduce the level of sensitivity required in a detection system. This may make more likely the development a stand-off alpha detector using UVC radioluminescence to indicate the presence of alpha contamination. It would require the engineering of a solution, which could be stand alone or utilise gas environments already found in some field conditions and could be part of a package of detection solutions, including such elements as lenses and bespoke, optimised electronics, which would make up the final detection system.

The increase in signal using a flow of Xe, which in all three groups of experiments increased the signal intensity by more than 50%, shows a much greater change than in any of the other gases. Saito et al. [16] found a peak at a wavelength of 175 nm which is just under the 185 nm sensitivity range of the UVTron, but as the peak has a full width half maximum (FWHM) of 15 nm there may be sufficient overlap into the UVTron detectable range. The shorter travel of alpha particles in Xe (25.2 mm at atmospheric pressure) [16] suggests that alpha deposits its energy closer to the alpha source. The main fluorescing area has been found to be within 10 mm of the alpha source [6]. Hence, this shorter mean free path may be instrumental in increasing the signal intensity close to the source and therefore aid location of the source by the UVTron.

Over the duration of the experiments, which were carried out for no less than and approximately for one hour, there was little variation in the measured cps despite a likely accumulation of the flowed gas inside the box. This indicates that the flow method may potentially be as useful for increasing radioluminescence as a purged atmosphere. As the area around the source which produces the most radioluminescence is within 10 mm of the source itself [6], hence it is the atmosphere in this area which most affects radioluminescence, which could conceivably be greatly modified by a flow of gas in the vicinity of the source, as these results would suggest.

Slight alteration in the set up due to removal and replacement of the equipment between experiment groups will account for some of the differences seen between the measurements for each of the gases, and hence an average percentage increase is included in Table 2. This variation is likely due to minor differences in distance between or the angle of the sensor relative to the source, indicating that this set up is sensitive to small variations which would need to be addressed in any field operative detector system.

The relatively small increase seen in an N_2_ gas flow may seem in conflict with N_2_ purged atmosphere results where there is seen an increase in radioluminescence. This likely due to the UVC wavelength range and a possible lack of NO which has been shown to cause radioluminescence in the UVC wavelength range in an N_2_ purged atmosphere [22]. The lack of NO produced by a flow as in comparison to a purge may be due to the lack of excited NO molecules being generated by the alpha particles in this circumstance.

Using Hurtgen et al.’s [25] method of calculation, the limit of detection ( $L_{d}$) was determined. This method was used as it is specifically designed for detectors with a low or zero background count, which the UVTron has in normal lab lighting conditions. The limit of detection is independent of the signal count, depending only on the background level, and is determined with a confidence level of 95.45% (as advised by ISO, 1993) through the equation

(1) $$at\;L_{d},\;s_{net}=2.86+4.78\sqrt{\left(b+1.36\right)}$$

where $L_{d}$ = the limit of detection, $s_{net}$ is the net signal (gross signal minus background), and b is the background count. For laboratory conditions, the limit of detection for these experiments was 17.4 net counts for the first set of experiment, and 15.3 net counts for the second and third sets of experiments for a count time of one hour (3600 s). The difference is due to small changes in background conditions, where the background counts averaged 7.9 and 5.4 counts, respectively, in 3600 s. As can be seen, the very low background count of the UVTron leads to a very low limit of detection in laboratory conditions. In comparison, the net counts with a source present ranged from 1223 counts in nitrogen to 3045 counts in xenon in one hour (3600 s). Based on the low detection limit, the system could easily be configured to alert for the presence of an alpha emitter based on a count rate above a set threshold, which is likely to be very low. As an example of the sensitivity of this sensor and off-the-shelf electronics, Hurtgen et al.’s [25] method was used to calculate the lowest activity which could have been detected here. In air, the minimum activity at this distance is between 89 and 149 kBq, which is similar to nitrogen at between 82 and 144 kBq. Xenon as expected has the lowest limit of detection at an activity of 47 kBq in the second and third set of experiments. Although certain applications would find this limit alone beneficial, with the aforementioned use of optics, optimised electronics, collimation, and other elements of a detector system based on this sensor, this limit could be extended significantly. Therefore, the experiments carried out to date would suggest that gas flows could be useful in field conditions to increase the signal and make detection easier.

Through further experimentation, it may be possible to determine the mechanism for luminescence in the UVC region, the third continuum, which may throw light on which gases are more likely to provide a suitable radioluminescence yield in this region and possibly lead to a predictive model of which gases are likely to fluoresce in this region for further testing. 

## Figures and Tables

**Table 1:** Table of gases showing flow rates and experiment groups.

Gas	Symbol	Purity	Approximate Flow Rate mL/min		
			Group 1 [13]	Group 2	Group 3
Nitrogen	N_2_	N5.0	65	65	65
Xenon	Xe	N5.0	50	65	65
P10	10% CH4/90% Ar	±5%	60	-	65
Krypton	Kr	N5.0	55	65	65
Neon	Ne	CP grade	40	-	65
Argon	Ar	N5.0	*-*	-	65

**Table 2:** Table of gas flow results.

Gas	Group 1	Group 2	Group 3	Average % Increase
	CPS [13]	CPS	CPS	
	(% incr)	(% incr)	(% incr)	
N_2_	0.34	0.4716	0.4898	(9.09)
	(3.61)	(14.86)	(8.79)	
Xe	0.5004	0.8431	0.8541	(82.5)
	(52.47)	(105.32)	(89.71)	
Ne	0.4131	-	0.6812	(38.69)
	(25.87)		(51.51)	
Kr	0.4045	0.6003	0.6247	(36.08)
	(23.26)	(46.21)	(38.77)	
P10	0.4339	-	0.5983	(32.55)
	(32.21)		(32.9)	
Ar	-	-	0.5865	(30.27)
			(30.27)	

## References

[1] Baschenko S.M. (2004). Remote optical detection of alpha particle sources. J. Radiol. Prot..

[2] Waldenmeir T. (2008). Spectral resolved measurement of the nitrogen fluorescence yield in air induced by electrons. Astropart. Phys..

[3] Sand J., Nicholl A., Hrnecek E., Toivonen H., Toivonen J., Perajarvi K. (2016). Stand-Off Radoluminescence Mapping of Alpha Emitters Under Bright Lighting. IEEE Trans. Nuclear Sci..

[4] Kume N., Takakura K., Nakayama K., Kuroda H., Izumi M., Mukai N. Remote Detector of Alpha-Ray Using Ultraviolet Ray Emitted by Nitrogen in Air. Proceedings of the Nuclear Science Symposium and Medical Imaging Conference (NSS/MIC).

[5] Inrig E., Koslowsky V., Andrews B., Dick M., Forget P., Ing H., Hugron R., Wong L. (2011). Development and testing of an air fluorescence imaging system for the detection of radiological contamination. AIP Conf. Proc..

[6] Sand J., Ihantola S., Perajarvi K., Nicholl A., Hrnecek E. (2015). Imaging of alpha emitters in a field environment. Nuclear Instrum. Methods Phys. Res. A.

[7] Sand J., Ihantola S., Perajarvi K., Toivonen H., Nicholl A., Hrnecek E., Toivonen J. EMCCD Imaging of Strongly Ionizing Radioactive Materials for Safety and Security. Proceedings of the 2013 Conference on Lasers & Electro-Optics Europe & International Quantum Electronics Conference (CLEO EUROPE/IQEC).

[8] Leybourne A.E., Creasey S., Dixon J., Lee J., Messer G., Neal S., Rayborn G.H., Speaks D., Stephens J., Strange T. Long range detection of radiation induced air fluorescence. Proceedings of the Institute of Nuclear Materials Management Annual Meeting.

[9] Sand J., Hannuksela V., Ihantola S., Perajarvi K., Toivonen H., Toivonen J. Remote Optical Detection of Alpha Radiation. IAEA, International Nuclear Information System, 2010 Ref: IAEA-CN-184/23. http://www.iaea.org/inis/collection/NCLCollectionStore/_Public/42/081/42081464.pdf.

[10] Hannuksela V., Toivonen J., Toivonen H., Sand J. Optical Remote Detection of Alpha Radiation. Proceedings of the Third European IRPA Congress.

[11] Sayre R., Dowdy J., Poh-Fitzpatrick M. (2004). Dermatological Risk of Indoor Ultraviolet Exposure from Contemporary Lighting Sources. Photochem. Photobiol..

[12] Ivanov I.P., Stepanov V.E., Smirnov S.V., Volkovich A.G. Development of Method for Detection of Alpha Contamination with Using UV-Camera “DayCor” by OFIL. Proceedings of the Nuclear Science Symposium and Medical Imaging Conference (NSS/MIC).

[13] Crompton A.J., Gamage K.A.A., Bell S., Wilson A.P., Jenkins A., Trivedi D. (2017). First Results of Using a UVTron Flame Sensor to Detect Alpha-Induced Air Fluorescence in the UVC Wavelength Range. Sensors.

[14] Morii H., Mizouchi K., Nomura T., Sasao N., Sumida T., Kobayashi M., Murayama Y., Takashima R. (2004). Quenching effects in nitrogen gas scintillation. Nuclear Instrum. Methods Phys. Res. Sect. A Accel. Spectrom. Detect. Assoc. Equip..

[15] Brett J., Koehler K.E., Bischak M., Famiano M., Jenkins J., Klankowski L., Prashantamani N., Pancella P., Lakis R. (2017). Spectral measurements of alpha-induced radioluminescence in various gases. Nuclear Instrum. Methods Phys. Res. A.

[16] Saito K., Tawara H., Sanami T., Shibamura E., Sasaki S. (2002). Absolute number of scintillation photons emitted by alpha particles in rare gases. IEEE Trans. Nuclear Sci..

[17] Strickler T.D., Arakawa E.T. (1964). Optical Emissions from Argon Excited by Alpha Particles: Quenching Studies. J. Chem. Phys..

[18] Saito K., Sasaki S., Tawara H., Sanami T., Shibmura E. (2003). Simultaneous measurement of absolute numbers of electrons and scintillation photons produced by 5.49 MeV alpha particles in rare gases. IEEE Trans. Nuclear Sci..

[19] Gray L. (1944). The ionisation method of measuring neutron energy. Math. Proc. Camb. Philos. Soc..

[20] Gibson G.E., Gardiner E.W. (1927). The Ionisation and Stopping Power of Various Gases for Alpha-Particles from Polonium: I. Phys. Rev..

[21] Jesse W.P., Forstat H., Sadauskis J. (1950). The ionisation in argon and air by the single alpha-particles as a function of their energy. Phys. Rev..

[22] Kerst T.H.G., Sand J., Toivonen J. Dynamic Enhancement of Radioluminescence in Solar Blind Spectral Region. Proceedings of the 2017 Conference on Lasers and Electro-Optics Europe & European Quantum Electronics Conference (CLEO/Europe-EQEC).

[23] Hurst G.S., Bortner T.E., Strickler T.D. (1968). Proton Excitation of the 1300-Å Continuum in Argon. J. Chem. Phys..

[24] UQG Optics, Data Sheet, UV Fused Silica—Spectrosil. http://www.uqgoptics.com/pdf/Fused%20Silica%20-%20Spectrosil1.pdfb.

[25] Hurtgen C., Jerome S., Woods M. (2000). Revisiting Currie—How low can you go?. Appl. Radiat. Isot..

